# Defining Falciparum-Malaria-Attributable Severe Febrile Illness in Moderate-to-High Transmission Settings on the Basis of Plasma *Pf*HRP2 Concentration

**DOI:** 10.1093/infdis/jis675

**Published:** 2012-11-07

**Authors:** Ilse C. E. Hendriksen, Lisa J. White, Jacobien Veenemans, George Mtove, Charles Woodrow, Ben Amos, Somporn Saiwaew, Samwel Gesase, Behzad Nadjm, Kamolrat Silamut, Sarah Joseph, Kesinee Chotivanich, Nicholas P. J. Day, Lorenz von Seidlein, Hans Verhoef, Hugh Reyburn, Nicholas J. White, Arjen M. Dondorp

**Affiliations:** 1 Mahidol-Oxford Tropical Medicine Research Unit, Faculty of Tropical Medicine, Mahidol University, Bangkok, Thailand; 2 Centre for Tropical Medicine, Churchill Hospital, University of Oxford, Oxford; 3 Department of Clinical Research; 4 MRC International Nutrition Group, London School of Hygiene and Tropical Medicine; 5 Medical Research Council, London, United Kingdom; 6 Cell Biology and Immunology Group, Wageningen University; 7 Laboratory for Microbiology and Infection Control, Amphia Hospital, Breda, the Netherlands; 8 Amani Centre; 9 Tanga Medical Research Centre, National Institute for Medical Research,Tanga; 10 Teule Hospital, Muheza, Tanzania; 11 Menzies School of Health Research, Casuarina, Australia

**Keywords:** case definition, severe malaria, *Plasmodium falciparum*, histidine-rich protein 2, malaria-attributable disease, asymptomatic parasitemia, bacteremia, Tanzania

## Abstract

***Background.*** In malaria-endemic settings, asymptomatic parasitemia complicates the diagnosis of malaria. Histidine-rich protein 2 (HRP2) is produced by *Plasmodium falciparum*, and its plasma concentration reflects the total body parasite burden. We aimed to define the malaria-attributable fraction of severe febrile illness, using the distributions of plasma *P. falciparum* HRP2 (*Pf*HRP2) concentrations from parasitemic children with different clinical presentations.

***Methods.*** Plasma samples were collected from and peripheral blood slides prepared for 1435 children aged 6−60 months in communities and a nearby hospital in northeastern Tanzania. The study population included children with severe or uncomplicated malaria, asymptomatic carriers, and healthy control subjects who had negative results of rapid diagnostic tests. The distributions of plasma *Pf*HRP2 concentrations among the different groups were used to model severe malaria-attributable disease.

***Results.*** The plasma *Pf*HRP2 concentration showed a close correlation with the severity of infection. *Pf*HRP2 concentrations of >1000 ng/mL denoted a malaria-attributable fraction of severe disease of 99% (95% credible interval [CI], 96%–100%), with a sensitivity of 74% (95% CI, 72%–77%), whereas a concentration of <200 ng/mL denoted severe febrile illness of an alternative diagnosis in >10% (95% CI, 3%–27%) of patients. Bacteremia was more common among patients in the lowest and highest *Pf*HRP2 concentration quintiles.

***Conclusions.*** The plasma *Pf*HRP2 concentration defines malaria-attributable disease and distinguishes severe malaria from coincidental parasitemia in African children in a moderate-to-high transmission setting.

Children <5 years old have the highest burden of malaria and malaria-associated mortality in sub-Saharan Africa [1–4]. In these moderate-to-high transmission areas, the diagnosis of severe malaria is challenging. Parasitemic children with severe febrile illness can suffer from severe malaria but the parasitemia can also be coincidental, with an alternative illness causing severe disease. This is because partial immunity develops early in life in regions of high malaria endemicity, and malaria parasites can be tolerated without development of symptoms [5, 6]. Community-based cross-sectional studies conducted in these settings typically show that >10% of children <5 years old are parasitemic by microscopy yet symptom free, with the prevalence varying by age, exposure to infection, transmission season, and other factors [7–10].

Commonly used case definitions of malaria rely on the presence of fever and detection of malaria parasites on peripheral blood films and thus lack specificity. In addition, symptoms of severe malaria are nonspecific and can have different etiologies [11–13].

More-accurate case definitions for clinical or severe malaria are required for clinical management and research purposes. The specificity of a malaria case definition can be improved by using a parasite density threshold based on peripheral blood parasitemia [7, 14, 15]. This approach is useful as an epidemiological tool, but it lacks accuracy for clinical management. Peripheral blood parasitemia does not represent the sequestered parasite burden, which is pivotal to the pathophysiology of severe falciparum malaria. Asexual parasites in the second half of the erythrocytic stage of the life cycle effectively adhere to the endothelial lining of microcirculation vessels, which prevents detection of these parasites in peripheral blood films [16].

*Plasmodium falciparum* histidine-rich protein 2 (*Pf*HRP2) is a parasite-derived water-soluble protein and is released in discrete amounts into the plasma, predominantly during schizont rupture [17]. Released P*f*HRP2 is distributed over the plasma volume and, therefore, the *Pf*HRP2 concentration in plasma reflects the total body parasite burden, including the sequestered parasites. Studies involving Asian adults [18, 19] and African children [20, 21] show that, in contrast with the peripheral blood parasite density, the plasma *Pf*HRP2 concentration correlates strongly with disease severity and outcome.

We hypothesized that the plasma *Pf*HRP2 concentration, as a measure of the total parasite burden determining disease severity, can be used to define malaria-attributable disease in malaria-endemic regions where coincidental peripheral blood parasitemia is common.

In this study, we compared the distribution of peripheral blood parasitemia versus the plasma *Pf*HRP2 concentration in healthy, rapid diagnostic test (RDT)-negative controls, in asymptomatic carriers, and in patients with uncomplicated or severe malaria and used this to estimate the malaria-attributable fraction of severe disease.

## METHODS

The study was conducted in the rural lowlands of northeastern Tanzania. Peripheral blood slides and plasma *Pf*HRP2 samples were collected in 1 community-based and 2 hospital-based studies in the neighboring districts of Handeni and Muheza, Tanga Region, that have similar intensities of malaria transmission [9, 22].

Four clinical severity groups were defined: patients with severe malaria, patients with uncomplicated malaria, asymptomatic carriers, and healthy control subjects with negative results of an RDT.

Cases of severe malaria were identified in patients from the hospital-based studies, using modified clinical World Health Organization criteria that were confirmed by positive results of a parasite lactate dehydrogenase (pLDH) RDT (OptiMAL-IT, DiaMed, Switzerland) and/or *Pf*HRP2-based RDT (Paracheck, Orchid Biomedical, India). Severity criteria included decreased consciousness (coma or severe prostration), convulsions, respiratory distress or acidotic breathing, shock, severe symptomatic anemia (hemoglobin concentration <5 g/dL), and hypoglycemia (glucose concentration <2.5 mmol/L) [23]. Children with uncomplicated malaria, asymptomatic carriers, and healthy controls were identified in the community-based study on the basis of results of a pLDH-based RDT (CareStart, Access Bio, United States). Uncomplicated malaria was defined by fever (axillary temperature ≥37.5°C), absence of severity criteria, and a positive result of a pLDH-based RDT [24, 25]. Asymptomatic carriers were defined as afebrile children (on the basis of their clinical history and an axillary temperature of <37.5°C at presentation) with a positive result of a pLDH-based RDT. Controls were afebrile children with negative results of a pLDH-based RDT.

In the community-based study, asymptomatic children were recruited between February and August 2008 in the context of the baseline screening for a randomized trial that assessed the effect of micronutrient supplementation on the incidence of uncomplicated malaria [25]. In 4 villages in Handeni District, all resident children aged 6–60 months were invited for the screening, and those with a height-for-age *z* score of ≤−1.5 SD, a weight-for-height *z* score of ≥−3 SDs, and a hemoglobin concentration of ≥7 g/dL were eligible to participate. Those who were unlikely to comply with interventions, whose parents/guardians refused to provide consent, or who had signs of severe or chronic disease on clinical examination were excluded.

**Figure 1.**
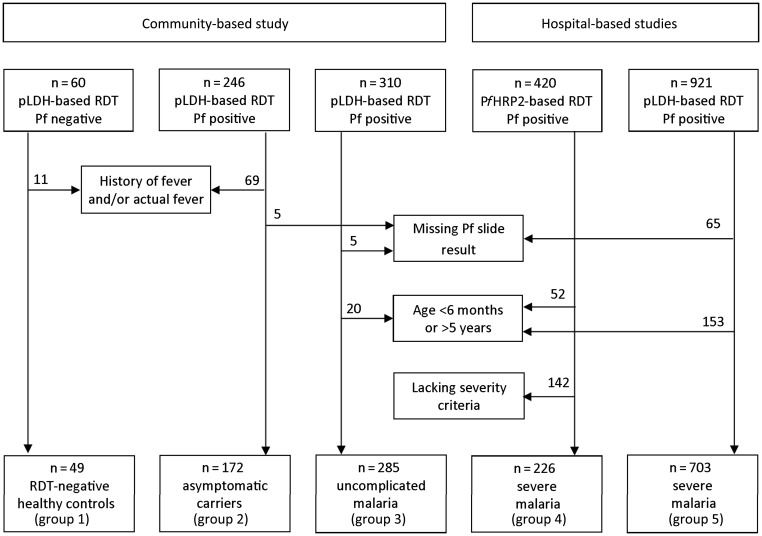
Selection of study subjects. Abbreviations: Pf, *Plasmodium falciparum*; *Pf*HRP2, *P. falciparum* histidine-rich protein 2; pLDH, parasite lactate dehydrogenase; RDT, rapid diagnostic test.

In total, 246 of 612 children had a plasma sample and a RDT positive for *P. falciparum*. Of these, 177 were afebrile on examination and reported absence of fever within the past 48 hours. Slide results were available for 172 asymptomatic individuals, who were included in the present study (termed “group 2”). All parasitemic children at baseline were treated with an effective antimalarial (artemether-lumefantrine). We selected the first 60 consecutively enrolled RDT-negative children as controls (termed “group 1”), of whom 11 were subsequently excluded because they had a history of or current fever. Uncomplicated malaria cases were detected during the follow-up period of the trial. Parents were requested to bring study children to the clinic if their child developed a fever or became unwell. Of these, 285 randomly selected febrile children with a positive result of a pLDH-based RDT (termed “group 3”) were included in the analysis (Figure 1).

Severely ill parasitemic patients originated from 2 consecutive studies conducted at Teule Hospital (Muheza, Tanzania). The details of these studies have been published elsewhere [26, 27]. The first study assessed the causes of fever in 3639 febrile children admitted from June 2006 through May 2007 [26]. From this cohort, patients who had pathogens isolated by blood culture and a RDT positive for falciparum malaria, plus a random sample of patients with RDT-positive severe malaria but a negative blood culture result, were included in the analysis (termed “group 4”; n = 226). The second severe malaria group (termed “group 5”) was part of a severe malaria treatment trial (AQUAMAT) conducted from February 2007 through July 2010 (n = 703) [27]. These subjects were also part of a separate report describing the prognostic value of plasma *Pf*HRP2 levels among 3826 children across all AQUAMAT study sites [21].

All 3 studies were approved by the Tanzania Medical Research Coordinating Committee. The community-based study was also approved by the Ethical Review Committee of Wageningen University. The hospital-based studies were also approved by the London School of Hygiene and Tropical Medicine and the Oxford Tropical Research Ethics Committee. In all studies, written informed consent was obtained from parents or guardians of each participating child.

Experienced microscopists at the National Institute of Medical Research Tanga laboratory in Korogwe, Teule Hospital (Joint Malaria Programme), and the Mahidol-Oxford Tropical Medicine Research Unit in Bangkok read the malaria slides; the latter institution was also responsible for quality control. Parasitemia (parasites/µL) was calculated from the thick film per 200 white blood cells (WBCs) and the actual WBC count or, if missing, assuming 8000 WBC/µL (count/200 WBC × 40) [28]. In the AQUAMAT study, parasitemia was calculated from thin film per 1000 red blood cells (RBCs) (count/1000 RBCs × 125.6 × Hct) [29, 30].

Plasma *Pf*HRP2 was assessed from freeze-thawed ethylenediaminetetraacetic acid plasma samples by a commercial sandwich enzyme-linked immunosorbent assay (ELISA) kit (Celisa, Cellabs, Sydney, Australia), according to the manufacturer's instructions, with minor modifications [18]. Reference plasma with a known *Pf*HRP2 concentration was used to construct standard curves. Concentrations in diluted plasma dilutions were determined in duplicate according to the linear segment of the standard curve. Positive cases were defined as those in which duplicate derived concentrations were in agreement (ratio, 0.5–2) and the optical density relative to background was >3 SDs of the average background based on all plates.

### Statistical Analysis

Data were analyzed with Stata, version 12 (StataCorp, United States). Parasite counts and *Pf*HRP2 concentrations were normalized by log_10_ transformation. Normally distributed or log_10_-normalized variables were compared using a Student *t* test, and the remainder were compared by the Wilcoxon rank sum test. *Pf*HRP2 concentrations between blood-culture-positive patients and blood-culture-negative patients were compared according to *Pf*HRP2 quintiles for patients with severe malaria (groups 4 and 5).

### Modeling *Pf*HRP2 Concentrations According to Diagnostic Group

**Figure 2.**
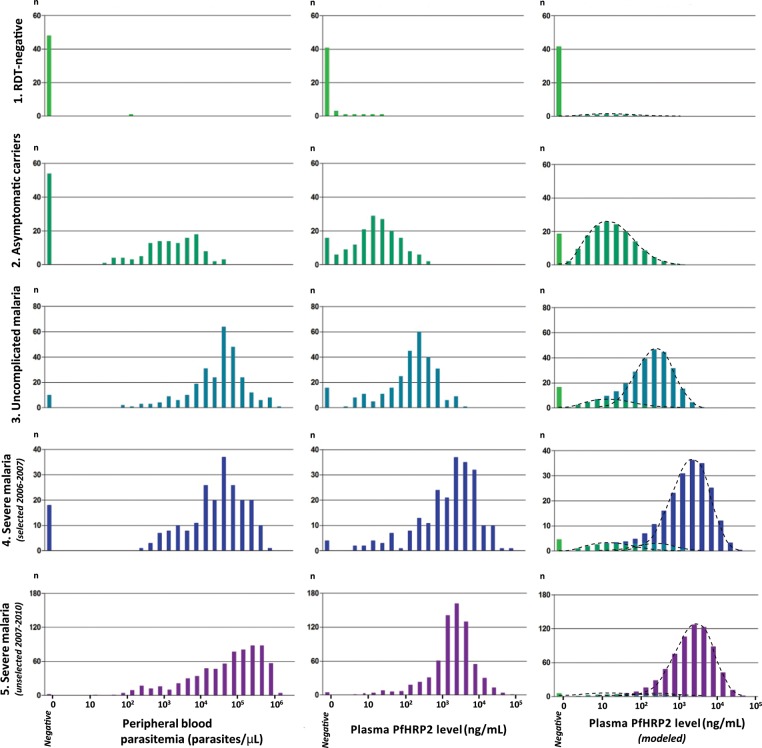
Frequency distributions of peripheral blood parasitemia, plasma *Plasmodium falciparum* histidine-rich protein 2 (*Pf*HRP2) concentrations, and modeled fitted *Pf*HRP2, according to malaria clinical group (1 = healthy rapid diagnostic test [RDT]–negative controls, 2 = asymptomatic carriers, 3 = uncomplicated malaria, 4 = severe malaria, 5 = severe malaria). The fitted *Pf*HRP2 distributions (right column) show the modeled *Pf*HRP2 distributions with the underlying contributing *Pf*HRP2 distributions of different diagnostic groups (dotted lines), composed of RDT-negative controls (light green), asymptomatic carriers (green), and patients with uncomplicated malaria (blue turquoise) or severe malaria (bright blue and purple).

Analysis of the observed *Pf*HRP2 concentrations suggested distinctive distributions according to severity of *P. falciparum* infection (Figure 2). In addition, the *Pf*HRP2 concentrations observed in patients with clinically defined severe malaria suggested contributions of underlying plasma *Pf*HRP2 distributions, as observed in RDT-negative controls, asymptomatic carriers, and patients with uncomplicated malaria (Figure 2), all representing severe illness with alternative causes. It was assumed that each diagnostic group (*k*) had a distinctive Weibull distribution of plasma *Pf*HRP2 concentrations and that the observed plasma *Pf*HRP2 distribution in the different clinical groups (*j*) was a composite of these Weibull distributions. The diagnostic groups (*k*) consisted of healthy controls (*k* = 1), asymptomatic carriers (*k* = 2), patients with uncomplicated malaria (*k* = 3), and patients with severe malaria (*k* = 4). The diagnostic groups of uncomplicated and severe malaria, in contrast with the clinically defined groups, exclude patients with coincidental parasitemia. A mechanistic model was constructed to infer the most likely Weibull distributions in each diagnostic group (*k*), described by the coefficients α_k_ and β_k_. The probability (*P*) that an individual (*i*) has a particular plasma *Pf*HRP2 concentration (*P*[*h_ji_*]) is then determined by the probability (*m_jk_*) that this individual belongs to diagnostic group *k*.
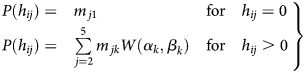

Two different groups with clinical severe malaria were included in the model, of which one was partly selected on the basis of the concomitant presence of bacteremia (group 4; see above). The model was used to define the plasma *Pf*HRP2-based malaria-attributable fraction in the unselected group of parasitemic patients with a clinical diagnosis of severe malaria (group 5). It differentiates severe malaria from the population with asymptomatic parasitemia and the population with uncomplicated malaria, who have severe disease of a different origin. The proportion of malaria-attributable disease (*y*), according to *Pf*HRP2 concentration (*h*), is given by
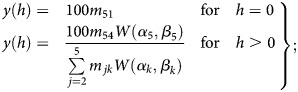
 that is, for each value of plasma *Pf*HRP2 concentration (*h*), the malaria-attributable fraction of severe disease is *m*_54_*W*(*α*_5_,*β*_5_) divided by the total number of individuals with the same *Pf*HRP2 concentration (*h*) predicted by the model as
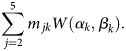
 The parameters were estimated by implementing a mixture model within WinBUGS [30]. Three chains were run, for a burn-in of 5000 iterations, followed by a further 5000 iterations to obtain posterior distributions. The model parameters were estimated with 95% credible intervals (CIs). Sensitivity was calculated using the model-derived number of patients with severe malaria as a reference.

## RESULTS

### Subject Characteristics

We analyzed data from 49 healthy RDT-negative controls (group 1), 172 children with asymptomatic parasitemia (group 2), 285 patients with uncomplicated malaria (group 3), and 226 patients (group 4) and 703 patients (group 5) with clinical severe malaria (Figure 1). Microscopy findings were negative in all RDT-negative controls, except for 1 child (145 parasites/µL). Baseline clinical and laboratory characteristics according to malaria clinical group are summarized in Table 1. Children with severe malaria were younger than children with uncomplicated malaria (*P* < .0001) or asymptomatic parasitemia (*P* < .0001) and also had lower hemoglobin concentrations (*P* < .0001). Admission characteristics and outcomes of patients with severe malaria (groups 4 and 5) are summarized in Table 2.

**Table 1.** Baseline Characteristics of the Study Population, According to Malaria Clinical Group

	Group 1: RDT-Negative Controls	Group 2: Asymptomatic Carriers	Group 3: Uncomplicated Malaria	Group 4: Severe Malaria	Group 5: Severe Malaria
Characteristic	(n = 49)	(n = 172)	(n = 285)	(n = 226)	(n = 703)
Female sex	20 (41)	91 (53)	141 (49)	125 (55)	339 (48)
Age, y	2.3 (1.5–3.6)	3.2 (2.3–4.1)	2.8 (1.9–4.0)	1.7 (1.1–2.6)	2.2 (1.2–3.1)
Weight-for-age *z* score^a^	−1.6 ± 0.7	−1.6 ± 0.7	NA	−1.5 ± 1.1	−1.1 ± 1.2
Temperature, °C	36.4 ± 0.4	36.5 ± 0.4	37.9 ± 1.3	38.0 ± 1.1	38.1 ± 1.0
Hemoglobin concentration,^b^ g/dL	11.3 (10.4–11.9)	10.3 (9.3–11.2)	9.8 (8.9–10.8)	4.8 (3.7–6.4)	6.5 (4.4–8.2)
Slide positive for *P. falciparum*	1 (2.0)	118 (68.6)	275 (96.5)	208 (92.0)	701 (99.7)
Parasitemia, parasites/µL					
Geometric mean (95% CI)	145	1602 (1189–2157)	29 836 (24 390–36 498)	28 187 (22 312–35 607)	46 619 (39 476–55 054)
Range	…	19–35471	96–1 448 094	221–626 431	16–1 375 069
*Pf*HRP2 concentration,^c^ ng/mL					
Geometric mean (95% CI)	4 (1–11)	19 (15–23)	163 (137–194)	1510 (1180–1933)	1746 (1577–1934)
Range	1–29	1–546	3–4343	4–87 199	5–56 818

Data are no. (%) of children, mean ± SD, or median (interquartile range), unless otherwise specified.

Abbreviations: CI, credible interval; NA, not available; *P. falciparum, Plasmodium falciparum; Pf*HRP2, *P. falciparum* histidine-rich protein 2; RDT, rapid diagnostic test.

^a^ Data were missing for 7 children in group 4 and 1 child in group 5.

^b^ Data were missing for 1 child in group 1, 3 children in group 2, and 2 children in group 5.

^c^ Data are for individuals with detectable concentrations (8 in group 1, 156 in group 2, 269 in group 3, 222 in group 4, and 698 in group 5).

**Table 2.** Admission Characteristics and Outcomes of Children With Severe Malaria

Variable	Group 4 (n = 226)	Group 5 (n = 703)
Coma (BCS ≤2 or GGS ≤10)	30 (13)	213 (30)
Prostration (inability to sit)	106 (47)	403 (57)
Convulsions (≥2 within 24 h)	40 (18)	268 (38)
Severe anemia (hemoglobin concentration <5 g/dL)	128 (57)	221 (32)
Hypoglycemia (glucose concentration <2.5 mmol/L)	27 (12)	145 (21)
Acidosis (lactate concentration >5 mmol/L or base excess ≤−8 mmol/L)^a^	97 (43)	314 (49)
Respiratory distress^b^	74 (33)	131 (19)
Shock^c^	21 (9)	111 (16)
Blood culture positivity^d^	47 (20.8)	36 (5.1)
Mortality	31 (13.7)	99 (14.1)

Data are no. (%) of children.

Abbreviations: BCS, Blantyre coma scale; GCS, Glasgow coma scale.

^a^ Data were missing for 29 children in group 4 and 61 children in group 5.

^b^ Defined as nasal alar flaring, costal indrawing, use of accessory muscles, or severe tachypnea.

^c^ Compensated shock (capillary refill time of ≥3 seconds or presence of a temperature gradient with systolic blood pressure of ≥70 mm Hg) and decompensated shock (systolic blood pressure of <70 mmHg) combined.

^d^ Data were missing for 3 children in group 5.

*Pf*HRP2 concentrations were detectable in 8 of 49 healthy pLDH negative controls (16%), 156 of 172 asymptomatic patients (91%), 269 of 285 patients with uncomplicated malaria (94%), and 222 of 226 patients (98%; group 4) and 698 of 703 patients (99%; group 5) with severe malaria (Table 1). The distributions of peripheral blood parasitemia and *Pf*HRP2 concentrations according to clinical groups are displayed in Figure 2. Plasma *Pf*HRP2 concentrations were associated with the severity of *P. falciparum* infection, whereas peripheral blood parasitemia was not.

### Plasma *Pf*HRP2-Based Malaria-Attributable Disease in Parasitemic Severe Febrile Illness

**Figure 3.**
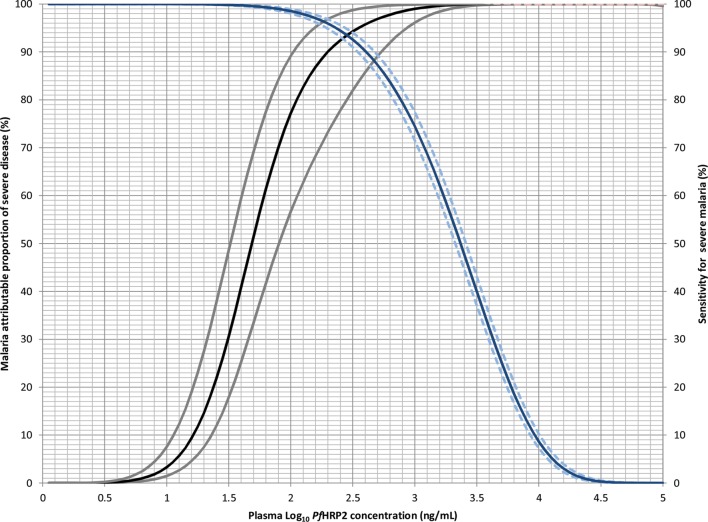
Malaria-attributable proportion (left axis) and sensitivity (median, 95% credible interval; right axis) for severe disease, according to log_10_ plasma *Plasmodium falciparum* histidine-rich protein 2 (*Pf*HRP2) concentration. The malaria-attributable proportion was derived from the predicted *Pf*HRP2 distributions from the median (95% credible interval) values of the *m_ij_* distributions of individuals in each malaria diagnostic group (see Supplementary Materials).

The observed *Pf*HRP2 distributions in the clinical groups were modeled as a composite of the *Pf*HRP2 distributions of the contributing diagnostic groups (Figure 2).The model-derived parameter estimates for *m_jk_* denoting the probability that an individual from clinical group *j* = 1–5 belongs to diagnostic groups *k* = 1–4 are given in the Supplementary Materials. From these parameter estimates, the predicted distributions were fitted to the observed distributions and used to derive malaria-attributable proportions according to the log_10_ plasma *Pf*HRP2 concentration in the unselected clinical group of severely ill parasitemic children (group 5; Figure 3). This shows that *Pf*HRP2 levels of >1000 ng/mL correspond to a malaria-attributable fraction of 99% (95% CI, 96%–100%), with a sensitivity of 74% (95% CI, 72%–77%). The proportion of malaria-attributable disease declined at lower *Pf*HRP2 concentrations. Below 200 ng/mL, an alternative diagnosis than malaria was suggested in >10% (95% CI, 3%–27%) of patients, whereas this proportion increased to >50% (95% CI, 31%–67%) at concentrations of <50 ng/mL.

### Blood Cultures

**Figure 4.**
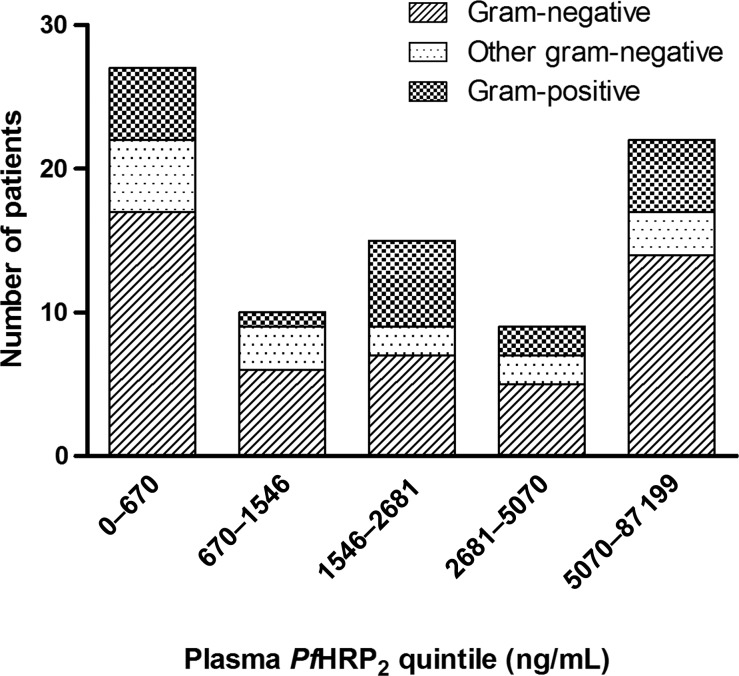
Blood culture positivity, according to plasma *Plasmodium falciparum* histidine-rich protein 2 (*Pf*HRP2) quintile, in patients with severe malaria. Gram-positive bacteria included *Streptococcus pneumonia, Staphylococcus aureus,* β-hemolytic *Streptococcus.* Other gram-negative bacteria included *Haemophilus influenza* (type b), unspecified gram-negative rods, *Salmonella typhi, Acinetobacter baumannii, Burkholderia cepacia, Kingella kingae, Neisseria* species*, Pseudomonas oryzihabitans,* and *Pasteurella* species*.* Gram-negative bacteria included *Salmonella* species*, Escherichia coli, Enterobacter cloacae,* and *Klebsiella* species. Contaminants included *Micrococcus* species*, Bacillus* species*,* coagulase-negative *Staphylococcus,* yeast, *Corynebacterium* species (diphtheroids), unspecified gram-positive rods*,* mixed bacterial species*, Ralstonia pickettii,* α-hemolytic *Streptococcus viridans, Sphingomonas paucimobilis, Pseudomonas stutzeri, Chryseomonas luteola,* and *Stenotrophomonas maltophilia*.

Blood culture results were positive among 83 patients with severe malaria (Table 2), and based on the sample selection criteria, this proportion was higher in group 4. Patients with a positive blood culture result were overrepresented in the lowest and highest plasma *Pf*HRP2 quintiles (Figure 4). Of 90 patients with a *Pf*HRP2 concentration below the threshold of 200 ng/mL, 16 (18%) had positive blood culture results.

## DISCUSSION

This study shows a clear stepwise increase in plasma *Pf*HRP2 concentrations according to disease severity from asymptomatic parasitemia, to uncomplicated malaria, to severe malaria. There was substantially less overlap in the distributions of plasma *Pf*HRP2 concentrations between groups as compared to the distributions of peripheral blood parasitemia. The distinct distributions between diagnostic groups enabled us to model the proportion of malaria-attributable disease on the basis of plasma *Pf*HRP2 level at admission and to distinguish this from patients with coincidental peripheral blood parasitemia in whom severe disease is caused by an alternative disease. The *Pf*HRP2-based model performed better than a previously described model that was based on peripheral blood parasitemia [15]. The proportion of malaria-attributable disease dropped to <50%, with a sensitivity of >99% at plasma *Pf*HRP2 concentrations of <50 ng/mL, in which case additional diagnostic tests are indicated to identify alternative diseases. The current model also accurately identified patients with a very high probability of severe malaria, with acceptable sensitivity. A threshold of 1000 ng/mL defined a population of patients with severe malaria not diluted by patients with coincidental parasitemia (<1%), which is mainly useful for defining a study population in a research setting, but is also useful for the treating clinician. A low plasma *Pf*HRP2 concentration in a parasitemic patient with severity signs should not result in withholding of treatment with antimalarials, but should prompt the treating physician to look for other possible diseases, depending on the clinical presentation and resources (eg, blood culture, lumbar puncture, chest radiograph, and computed tomography of the cerebrum). In African settings, were diagnostic facilities are scarce and treatment stock-outs occur, the *Pf*HRP2 concentration can also help to prioritize these resources.

A previous study reported the strong prognostic significance of plasma *Pf*HRP2 level for death in a large cohort of African children with severe malaria and modelled the malaria attributable fraction in fatal cases [21]. The current study enabled a more accurate definition of the probability of nonmalarial disease at low plasma *Pf*HRP2 concentrations by incorporating children with asymptomatic parasitemia and uncomplicated malaria. It is reassuring that the identified plasma *Pf*HRP2 thresholds denoting high or low probabilities of alternative disease were highly consistent between these studies, which used different modeling techniques.

Our findings are supported by 2 recent studies involving African children. A small study among Tanzanian children showed a mean *Pf*HRP2 value of 1008 ng/mL in patients with cerebral malaria, compared with a *Pf*HRP2 concentration of 443 ng/mL in patients with uncomplicated malaria [20]. The diagnostic potential of the plasma *Pf*HRP2 concentration in pediatric cerebral malaria was also confirmed in a Malawian study, where the presence of malarial retinopathy was used as the reference test [32]. In contrast, 2 other studies in moderate-to-high transmission settings reported that the *Pf*HRP2 concentration does not reflect severity in children. In Papuan children, the median *Pf*HRP2 concentrations in uncomplicated and severe malaria were similar (584 vs 456 ng/mL) [33]. However, the case-fatality rate in the severe malaria group was <1%, suggesting moderately severe malaria in accordance with the low *Pf*HRP2 concentrations reported. A small study involving 22 Kenyan children with severe malaria reported low median *Pf*HRP2 concentrations of 63 ng/mL with absence of decay over 48 hours, which could be related to problems in the *Pf*HRP2 assay [34].

The prognostic usefulness of plasma *Pf*HRP2 concentration is in line with previous reports involving adult populations. A study among Thai adults showed a similar stepwise increase in plasma *Pf*HRP2 levels that was associated with disease severity [18]. In Indonesian adults, the mean *Pf*HRP2 value among patients with severe malaria was 1863 ng/mL, compared with 314 ng/mL among patients with moderately severe malaria [19]. In both studies, the plasma *Pf*HRP2 concentration was prognostic for a fatal outcome.

This is the first study to assess *Pf*HRP2 concentrations in healthy asymptomatic children in a moderate-to-high transmission area. Parasite densities that can be tolerated without causing symptoms vary substantially between individuals of different age groups, transmission intensities, and seasons [10, 15, 35, 36]. In moderate-to-high transmission settings, children aged <5 years represent a heterogeneous group with regard to levels of immunity. This is reflected by the younger age of children with severe malaria and by the older age of asymptomatic children, of whom 13 of 172 (8%) had parasite densities of >10 000 parasites/µL. Similarly high parasite densities have been reported in cross-sectional surveys in settings with moderate-to-high malaria transmission [15, 35]. The accuracy of *Pf*HRP2 concentration thresholds for defining malaria-attributable disease will vary with the level of acquired immunity in the population, because this factor determines the relative sizes of populations with asymptomatic parasitemia, compared with populations with uncomplicated or severe malaria, and thus determines the corresponding overlap of plasma *Pf*HRP2 distributions. The model prediction as a function of transmission intensity will be explored in a separate study. In addition, the prevalence of bacteremia will also affect the size of the population of individuals who have asymptomatic parasite infections or uncomplicated malaria but present with severe illness. Indeed, in the current study, selection of patients with a positive blood culture result (group 4) resulted in a relatively higher proportion of parasitemic patients with severe illness due to diseases other than malaria.

Detection of malarial retinopathy by fundoscopy is an alternative diagnostic tool that has been evaluated for its ability to distinguish children with cerebral malaria from encephalopathic children with coincidental parasitemia [37–40]. In the African setting, this has only been evaluated in comatose patients and requires considerable expertise and training and appropriate equipment. In comparison, the plasma *Pf*HRP2 concentration is positively associated with the entire clinical severity spectrum of *P. falciparum* infection. In this study, plasma *Pf*HRP2 was assessed by a quantitative ELISA. Our findings call for the development of a low-cost semiquantitative rapid test for the detection of plasma *Pf*HRP2 at suitable thresholds.

Positive blood culture results, particularly those for gram-negative organisms, were overrepresented among patients within the lowest and highest *Pf*HRP2 concentration quintiles. Blood cultures are known to have a limited sensitivity (around 40%) for detecting bacteremia [41]. The actual number of bacteremic patients could thus be 2.5-fold higher than detected, implying an actual proportion of bacteremic patients close to 50% among patients with a plasma *Pf*HRP2 concentration of <200 ng/mL (2.5 times the observed proportion of 18%). This would be consistent with results from a Malawian autopsy series, in which invasive bacterial infection was reported as the cause of death in 4 of 7 parasitemic patients (64%) with an alternative diagnosis [42]. Positive blood culture results for patients with high *Pf*HRP2 concentrations indicate concomitant bacteremia during severe malaria. There are several mechanisms that may explain this high rate of concomitant bacteremia, including a reduction in gut barrier function due to intense sequestration [43], which facilitates translocation of gut bacteria, or general immunosuppression due to macrophagocytic dysfunction induced by hemozoin and heme-oxygenase 1 [44–46]. Severe malarial anemia is particularly associated with invasive bacterial disease, mainly non-typhi *Salmonella* bacteremia [47]. *P. falciparum* infection predisposes to gram-negative bacteremia and can account for more than half of invasive bacterial disease in malaria-endemic areas [48]. Our data show that bacteremia contributes to severe illness but also occurs concomitantly in patients with severe malaria, warranting the use of broad-spectrum antibiotics in addition to prompt antimalarial treatment, preferably with parenteral artesunate.

The current study has several limitations. This is a retrospective analysis of pooled data sets. Patients with severe malaria in group 5 were also included in a previous publication on the prognostic value of *Pf*HRP2 concentration. Patients with severe malaria in group 4 were partly selected on the basis of blood culture positivity. However, patients were selected on the basis of clinical criteria and RDT results and not on the basis of *Pf*HRP2 concentrations, and the *Pf*HRP2 distributions in both severe malaria groups were similar. In patients with low parasitemia, the sensitivities of the peripheral blood slide and the RDT are relatively low, which could have affected the composition of the clinical groups.

In conclusion, our study shows that the plasma *Pf*HRP2 concentration can be used to estimate the proportion of malaria-attributable disease in African children in moderate-to-high transmission settings and can distinguish severe malaria from severe febrile illness with coincidental peripheral blood parasitemia. Bacteremia is prominent among patients with severe illness and low plasma *Pf*HRP2 concentrations, suggesting that malaria may not be their primary diagnosis. Bacteremia is also more frequent among patients with high plasma *Pf*HRP2 concentrations, denoting concomitant sepsis with severe malaria, which implies that administration of antibiotics is warranted for all patients with a clinical diagnosis of severe malaria.

## Supplementary Data

Supplementary materials are available at *The Journal of Infectious Diseases* online (http://jid.oxfordjournals.org/). Supplementary materials consist of data provided by the author that are published to benefit the reader. The posted materials are not copyedited. The contents of all supplementary data are the sole responsibility of the authors. Questions or messages regarding errors should be addressed to the author.
